# 

**Published:** 1917-02

**Authors:** 


### ANNOUNCEMENTS

WEST VIRGINIA STATE SOCIETY.—Will hold its annual meeting this year in Fairmont, April 11 to 13th.

MICHIGAN STATE DENTAL SOCIETY. This Society will hold its annual meeting in Detroit, April 12 to 14, 1917. Address Dr. C. G. Bates, Durand, Mich., for particulars.

TENNESSEE STATE DENTAL ASSOCIATION. The Golden Jubilee meeting of this Society will be held in Memphis, June 19th to 22d. The officers promise the most attractive program ever presented in the south. Social features will be prominent. All ethical dentists are invited to come and bring their wives.

D. M. CATTELL, *President.*  C. E. HINES, *Secretary.*

CARR CASE DECIDED. The Supreme Court of Illinois has reversed the decision of the lower courts which convicted C. M. Carr and J. J. Kitteringham of practicing dentistry illegally, because they were operating a school of instruction in Chicago for the treatment of pyorrhoea alveolaris.

